# BioPyC, an Open-Source Python Toolbox for Offline Electroencephalographic and Physiological Signals Classification

**DOI:** 10.3390/s21175740

**Published:** 2021-08-26

**Authors:** Aurélien Appriou, Léa Pillette, David Trocellier, Dan Dutartre, Andrzej Cichocki, Fabien Lotte

**Affiliations:** 1 Inria Bordeaux Sud-Ouest, 33405 Talence, France; lea.pillette@inria.fr (L.P.); david.trocellier@inria.fr (D.T.); dan.dutartre@inria.fr (D.D.); fabien.lotte@inria.fr (F.L.); 2 LaBRI (University of Bordeaux, CNRS, Bordeaux-INP), 33400 Talence, France; 3 RIKEN, Wako 351-0106, Japan; A.Cichocki@skoltech.ru; 4 Flit Sport, 33000 Bordeaux, France; 5 Skoltech, 143026 Moscow, Russia

**Keywords:** brain–computer interfaces (BCI), electroencephalography (EEG), Python platform, signal processing, machine learning, physiological signals

## Abstract

Research on brain–computer interfaces (BCIs) has become more democratic in recent decades, and experiments using electroencephalography (EEG)-based BCIs has dramatically increased. The variety of protocol designs and the growing interest in physiological computing require parallel improvements in processing and classification of both EEG signals and bio signals, such as electrodermal activity (EDA), heart rate (HR) or breathing. If some EEG-based analysis tools are already available for online BCIs with a number of online BCI platforms (e.g., BCI2000 or OpenViBE), it remains crucial to perform offline analyses in order to design, select, tune, validate and test algorithms before using them online. Moreover, studying and comparing those algorithms usually requires expertise in programming, signal processing and machine learning, whereas numerous BCI researchers come from other backgrounds with limited or no training in such skills. Finally, existing BCI toolboxes are focused on EEG and other brain signals but usually do not include processing tools for other bio signals. Therefore, in this paper, we describe BioPyC, a free, open-source and easy-to-use Python platform for offline EEG and biosignal processing and classification. Based on an intuitive and well-guided graphical interface, four main modules allow the user to follow the standard steps of the BCI process without any programming skills: (1) reading different neurophysiological signal data formats, (2) filtering and representing EEG and bio signals, (3) classifying them, and (4) visualizing and performing statistical tests on the results. We illustrate BioPyC use on four studies, namely classifying mental tasks, the cognitive workload, emotions and attention states from EEG signals.

## 1. Introduction

Research on brain–computer interfaces (BCIs) started in 1973 with Jacques Vidal and his concept of direct brain–computer communication [1], enabling the transfer of information from the human brain to a machine via brain signals, usually measured using electroencephalography (EEG) [2]. The main objective of BCIs is to enable people with severe motor impairments to send commands to a wheelchair, e.g., by imagining left or right hand movements to make the wheelchair turn left or right [3]. Such BCIs are called active BCIs since users are actively sending commands to the system, here a wheelchair, by performing mental imagery tasks [4]. Another type of BCIs proved particularly promising for human–computer interaction (HCI): passive BCIs [4]. Such BCIs are not used to directly control some devices, but to monitor in real time users’ mental states, e.g., mental workload or attention, in order to adapt an application accordingly. Some other BCIs do not use brain signals only. They can use a different type of input in addition, and are thus called hybrid BCIs [5]. This additional input could be physiological signals, such as heart rate (HR), electrodermal activity (EDA), breathing or signals from other external device, such as an eye-tracking system.

### 1.1. Motivation

Although promising, non-invasive BCIs are still barely used outside laboratories, due to their poor robustness with respect to noise and environmental conditions. In other words, they are sensitive to noise, outliers and the non-stationarity of electroencephalographic (EEG) signals [6,7]. While considering and optimizing user training is essential for improving such systems [8,9], the computing machinery considerably evolved in the last decades, and numerous signal processing and machine learning algorithms for brain signals classification have also been developed [10]. It is, therefore, important to be able to test many promising new algorithms, resulting from both signal processing and machine learning research, on different data sets related to different paradigms in order to identify the most efficient tools for their future uses. This type of study can be complicated to set up online because of the costs in terms of time, e.g., equipping a subject with different sensors to measure the activity in order to analyze it online, or in terms of calculation, given that some algorithms, such as deep learning, may require a lot of computing resources and would be difficult to run online. However, such studies are possible offline, by applying recent and promising classification algorithms on existing data sets, and are actually widely used in the BCI community, notably to compare various algorithms on the exact same data [11]. While offline studies are simpler to set up than online analyses, they still require specific tools and skills to be run effectively and efficiently. Indeed, using such algorithms requires expertise in programming (e.g., in MATLAB or Python), signal processing, machine learning, as well as statistics for analyzing the resulting performances of the different algorithms; however, many BCI researchers come from diverse backgrounds, such as cognitive science, neuroscience or psychology, and might not master all those skills. If multiple BCI toolboxes are already available, they all require such skills, and most of them are focused on EEG and other brain signals, but usually do not include processing tools for other biosignals. It therefore highlights the need for convenient toolboxes that would be free, open source and equipped with a graphical interface that would allow users to process and classify EEG and other biological signals offline without any programming skills.

### 1.2. Approach

**Figure 1:**
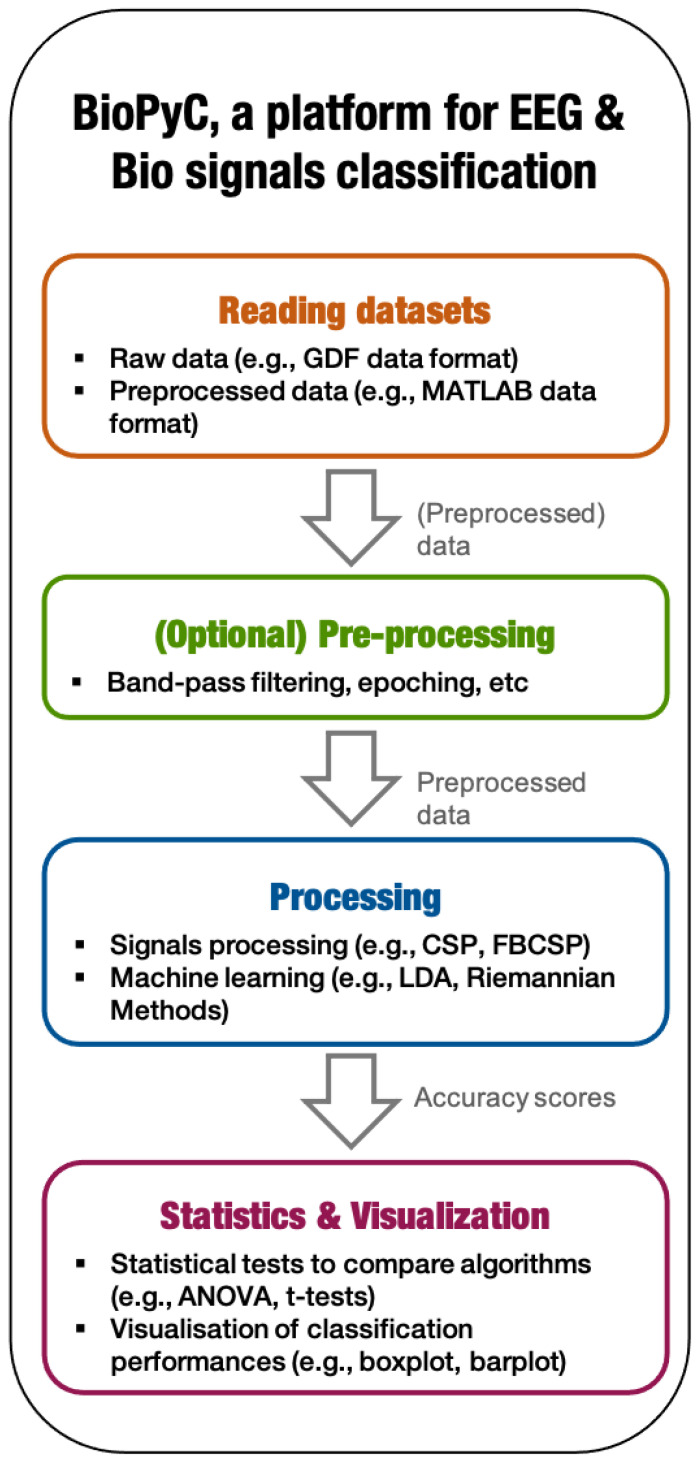
BioPyC data flow: the 4 main modules allow users to follow the standard BCI process for offline EEG and biosignal processing and classification.

We developed and tested BioPyC, an open-source and easy-to-use software for offline EEG and physiological signal processing and classification. BioPyC is free of charge, permissively licensed AGPL ( https://choosealicense.com/licenses/agpl-3.0/; accessed on 25 August 2021) and written in Python [12], an open-source programming language that is not only backed by an extensive standard library, but also by vast additional scientific computing libraries. This toolbox allows users to make offline EEG and biosignals analyses, i.e., to apply signal processing and classification algorithms to neurophysiological signals, such as EEG, electrocardiographic (ECG) signals, EDA or respiratory signal. See https://gitlab.inria.fr/biopyc/BioPyC/; accessed on 25 August 2021. In order to facilitate those analyses, BioPyC offers a graphical user interface (GUI) based on Jupyter [13] that allows users to handle the toolbox without any prior knowledge in computer science or machine learning. Finally, with BioPyC, users can apply and study algorithms for the main steps of biosignals analysis, i.e., pre-processing, signal processing, classification, statistical analysis and data visualization, as Figure 1 summarizes.

BioPyC enables users to work on two types of data sets: either raw data sets, which require the subsequent use of the pre-processing module (described below), or pre-processed data sets, which allow users to directly apply signal processing and machine learning algorithms on them. The pre-processing module of BioPyC offers basic features, i.e., band-pass filtering, cleaning and epoching raw EEG signals. Next, for the processing step, BioPyC offers two modules that enable users to use signal processing tools, such as spatial filters and feature extraction, but also machine learning algorithms for the classification of neurophysiological signals. Finally, another module enables users to automatically apply appropriate statistical tests on the obtained classification performances, to compare algorithms, and to obtain visualization plots describing those performances.

While some of the popular existing toolboxes, such as MOABB [11], pyRiemann [14] or MNE [15], listed in the “state-of-the-art” section below, have features in common with BioPyC, e.g., programming language or supported operating systems, as well as modules of the above-mentioned BCI process, e.g., signal processing or data visualization, none of them allow users to apply and study signal and classification algorithms for both EEG and physiological signals. BioPyC is indeed the first one which allows to study and compare classification algorithms for neurophysiological signals (at the moment EEG, EDA, respiration and ECG), offering modules for all the steps of this offline (hybrid) BCI and/or physiological computing process. Concerning the statistics, both tests and visualization for comparing classification algorithms performances are done automatically by BioPyC, again facilitating and speeding up users’ analyses: to the best of our knowledge, no other toolbox offers automatic statistical testing.

This paper is organized as follows: in Section 2, we present the state of the art of BCI platforms, the different features they offer, and conclude on the distinctive features that make BioPyC unique. In Section 3, we describe BioPyC modules and the data flow in more details, including the different algorithms that were implemented for the processing and classification of both EEG and biosignals, as well as the statistical tests available. Then, in Section 4, we present results of multiple research studies that were conducted using BioPyC so far, to illustrate its use and usefulness. On the one hand, BioPyC was applied to a widely used mental task data set, i.e., the “BCI competition IV data set 2a” [16]. On the other hand, we used it for studying different users’ mental states, e.g., cognitive workload, emotions and attention states. Finally, the discussion and conclusion come in Section 5, followed by the future works and improvements from which BioPyC could benefit in Section 6.

## 2. State-of-the-Art BCIs Platforms

**Figure 2:**
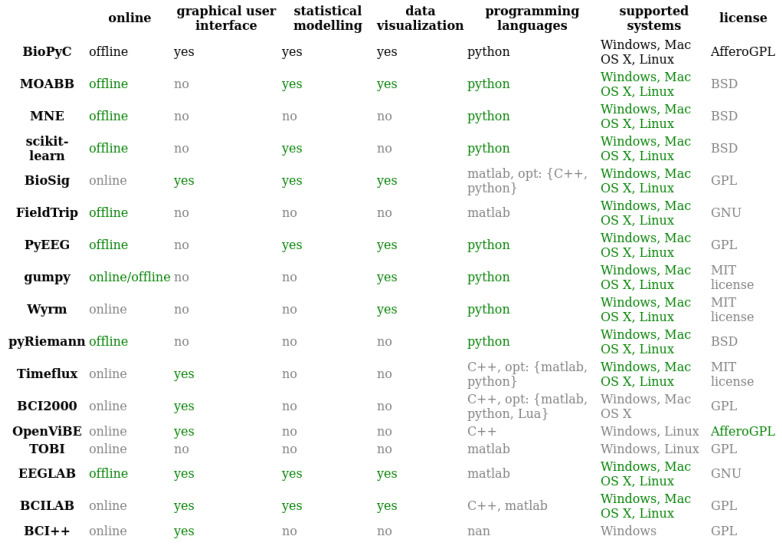
Comparison of main features of existing toolboxes having modules for EEG signals processing and classification. BioPyC values for each feature are written in black; values of features that are similar to those of BioPyC are written in green; and finally, values of features that differ from those of BioPyC are written in grey. “opt” stands for “optional” in the figure.

So far, several platforms for online experiments (i.e., BioSig [17], Timeflux [18], BCI2000 [19], OpenViBE [20], TOBI [21], BCILab [22] or BCI++ [23]) and also for offline studies (i.e., MOABB, MNE, EEGLAB [24], PyEEG) and finally for both online and offline studies (i.e., FieldTrip [25], Gumpy [26]) have been developed for researchers in order to build setups that would best suit their needs. They all have modules dedicated to the various BCI processing steps: data acquisition, signal processing, classification, statistical hypothesis testing and visualization [27]. We synthesized in Figure 2 the features of each of these existing platforms, i.e., online vs. offline studies, the availability of a graphical user interface (GUI), the existence of modules for statistical testing or data visualization, the programming language, and the supported systems as well as the type of license, in order to list the strengths and weaknesses of each one. Note that we presented both EEGLAB, which is made for EEG signals analysis, and the BCILAB toolbox, which is a plugin of EEGLAB made for online and offline classification and BCI, in this table. In the following, we compare the different platforms based on each of these features.

### 2.1. Graphical User Interface (GUI)

As explained above, it is useful to develop toolboxes with GUIs for the BCI community, as many BCI researchers do not come from a computer science background, notably cognitive scientists, neuroscientists or psychologists. In Figure 2, all Python-based toolboxes, i.e., MOABB [11], MNE [15], PyEEG [28], pyRiemann [14], gumpy [26] and Wyrm [29], suffer from the lack of such interfaces.

### 2.2. EEG Signal Processing

All platforms on Figure 2 naturally have an EEG signal processing system for classification, more or less elaborated, based on three classical steps, i.e., pre-processing, signal processing and classification.

#### 2.2.1. Pre-Processing

This step consists, for example, of band-pass filtering raw data into specific frequency bands, epoching raw data into trials or removing artifacts, among others. While all toolboxes propose some forms of pre-processing, the ones with the most advanced pre-processing tools, including plenty of methods dedicated to BCI, are MNE and BCILAB.

#### 2.2.2. Signal Processing

This step allows users to apply spatial or temporal filters on the signals and to extract features from them. Most existing platforms offer such filters and basic features, e.g., the common spatial pattern (CSP) filter or band power features, which are widely used for EEG signal analysis [30].

#### 2.2.3. Classification

All platforms also offer machine learning algorithms, from the simplest ones, such as linear discriminant analysis (LDA) [10] for most of them, to more complex ones, such as Riemannian geometry classifiers [31] for pyRiemann [14] and MOABB [11], deep Learning for gumpy [26] and various feature extraction methods in PyEEG [28].

### 2.3. Statistical Modeling and Data Visualization

The last step is divided between the statistical analysis and the visualization of performance results obtained by the machine learning algorithms. First, the visualization allows users to obtain graphs in order to have an overview of the classification results. Statistical modeling, on the other hand, consists of using statistical tests to compare the classification performances of the machine learning algorithms. This step is of primary importance when comparing classification algorithms since statistical tests allow to define, for a given study, which algorithm is the most likely to recognize patterns in neurophysiological signals. To the best of our knowledge, none of the platforms for offline signals analysis listed in Figure 2 have such dedicated features: they all require external toolboxes to do so but BioPyC does not: it provides embedded and automatic statistical analysis of the classification results.

### 2.4. Programming Languages

Another important criteria for defining a software is the programming language used, which can possibly make it easier or harder to develop new modules for it. Concerning the main BCI platforms, the programming languages that are used are MATLAB [32], C++ [33] and Python [12]. First, the proprietary programming language MATLAB is well known by the research community and widely used in laboratories, due to its popular rapid prototyping environment. However, the license is not free of charge nor always distributed to universities and laboratories. Second, C++ is free, very efficient, but difficult to use and, therefore, generally used by computer scientists and engineers only. Finally, Python is free, simple and extendable by non-computer scientists, making the prototyping and implementation of new modules for Python-based BCI platforms easier. Moreover, Python is widely used by the scientist community, i.e., machine learning experts, engineers and neuroscientists, and many machine learning libraries were implemented using this language, e.g., Scikit-learn [34], TensorFlow [35] or PyTorch [36].

### 2.5. Supported Systems

If BCI2000 [19], OpenViBE [20], TOBI [21] and BCI++ [23] do not support all operating systems, i.e., Windows, Mac OS X and Linux, all other platforms, i.e., BCILAB [22], pyriemann [14], Wyrm [29], gumpy [26], FieldTrip [25], pyEEG [28], BioSig [17], MNE [15] and MOABB [11] do.

### 2.6. Licenses

In the case of the main platforms presented in Figure 2, all of them are open source and have adopted either the general public license (GPL), lesser general public license (LGPL), Affero general public license (AGPL), Berkeley software distribution (BSD) or MIT license as their license.

### 2.7. Distinctive Features of BioPyC

BioPyC differs from several existing BCI platforms, due to the use of Python as its programming language. This difference should be highlighted, as Python is free of charge—compared to MATLAB, which is not—and simple and extendable by non-computer scientists, whereas C++ requires deep engineering skills. However, several BCI platforms, such as MOABB, MNE, PyEEG, pyRiemann, gumpy and Wyrm, are also implemented in Python but only MOABB offers algorithms for the full offline EEG signal classification process, i.e., pre-processing, signal processing and classification. This last feature makes MOABB closely resemble BioPyC. However, those two platforms differ in three main ways: (1) BioPyC comes with a GUI based on Jupyter notebook [13], which acts as a tutorial and allows users to interact with the toolbox in a guided way without requiring any programming nor programming skills, whereas MOABB and other Python-based platforms do not offer any GUI, and thus require programming by the user. Those existing platforms are thus most likely not usable by many BCI researchers coming from diverse backgrounds, such as cognitive science, neuroscience or psychology, with possibly no programming background; (2) MOABB allows users to perform an offline analysis only after having shared their data sets in open source, whereas BioPyC users can analyze their data sets on their own. It is an important feature to point out because most BCI researchers would want to analyze their own data before sharing them in open source; and (3) BioPyC offers modules for both statistical testing and visualization for comparing classification algorithms performance, and enables convenient analysis since tests and plots are chosen and applied automatically by the software, based on the data characteristics.

More generally and more importantly, BioPyC enables its users to classify physiological signals—i.e., HR, breathing, and EDA—in addition to EEG signals, whereas, with the exception of Biosig, no other BCI platform offers an algorithm to process and classify this type of physiological signal.

In conclusion, BioPyC distinguishes itself from other platforms through features that make it easy to use and more versatile. Indeed, it is based on Python and uses a Jupyter-based GUI. It also offers automatic statistical testing and visualization of classification results, as well as tools for classification of physiological signals such as as HR or breathing.

## 3. Materials and Methods

BioPyC comprises four main modules, allowing users to follow the standard BCI process for offline EEG and biosignals classification: (1) reading multiple neurophysiological data formats, (2) pre-processing, filtering and representing EEG and biosignals, (3) classifying those signals, and (4) performing visualization and statistical testing on the classification performance results. Users can follow these steps through a GUI based on Jupyter [13] and Voilà (https://blog.jupyter.org/and-voil%C3%A0-f6a2c08a4a93; accessed on 25 August 2021) that acts as a tutorial, explaining in a detailed way the actions to make at each step, highlighting the modularity of the platform. In this section, we detail the functionality of the platform Jupyter-based GUI, i.e., which tools we used to design it and how users are guided to interact with it. Then, we describe the modularity of BioPyC, i.e., how users can add any new module that may be necessary for their study. Finally, we present the different modules offered by BioPyC, corresponding to the major steps of the offline EEG and biosignals classification process.

### 3.1. Jupyter Notebook and Voilà as a GUI

**Figure 3:**
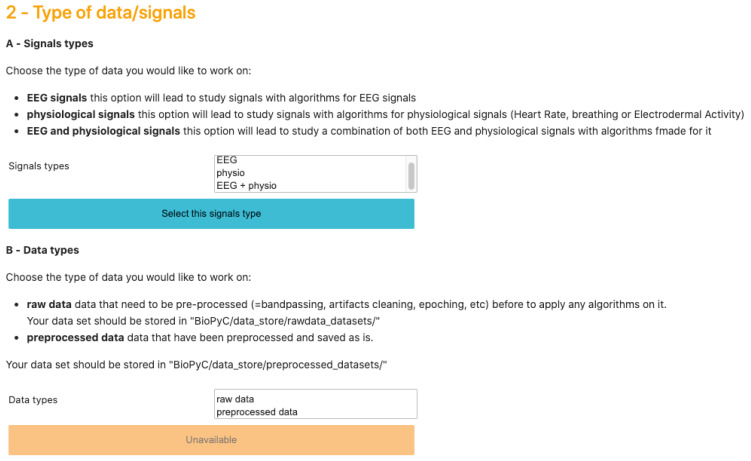
Screenshot of BioPyC’s widgets, i.e., “select multiples” and buttons at the step of selecting the type of data/signals to work on. In BioPyC, a blue button stands for the action to make, when the disabled orange ones stand for future actions to make: orange buttons turn blue when the previous action is done.

Jupyter notebook is a scientific notebook application that allows the user to write and execute code, as well as to view and save the results. With this tool, users can also write rich-text documentation, using Markdown formatting, and can display different widgets, such as textbox, checkbox or “select multiple” as we can see on Figure 3, to make options selections easier. Moreover, all these features are available in a single file that is accessed via a web browser. We then use voilà that turns Jupyter notebooks into standalone web applications in order to hide the code, and propose a seamless interface to users.

We designed this Jupyter interface in order to give users an intuitive path through the BCI process. Each step requires a choice from the user, and the options displayed in the following steps are presented according to past choices. For example, if a user chooses to work on pre-processed data, only data sets in which data were previously pre-processed will be displayed.

### 3.2. BioPyC Modularity

A strength of BioPyC is its modularity. Whereas the platform already comes with multiple existing modules that users can select with simple clicks, it is also possible to extend it by integrating new scripts as new modules. The kernel of the platform is designed to make such extensions easy: (1) store the new script in the appropriate folder, e.g., “BioPyC/src/classifiers/” for a new classifier or “eeg_contest/src/data_readers/” for a new data format reader, corresponding to a specific format (e.g., “.gdf” or “.mat”); (2) name the Python script after the classifier/data reader name with the “.py” extension; and (3) follow the class and method formalism that was used for other files of these modules.

### 3.3. Reading Data Sets

BioPyC offers users various data formats that they can work with: (1) starting with raw data, directly obtained with a data acquisition software, such as GDF [37]: this will lead to the optional pre-processing step; and (2) starting with pre-processed data, where trials from various runs and sessions have already been concatenated, where the data may have been cleaned with artifact removal, band-pass filtered and epoched. Users can also choose the type of signals they want to work on, i.e., EEG signals, physiological signals or a combination of EEG and physiological signals. This step is presented on Figure 3.

#### 3.3.1. Raw Data

The raw data are read and pre-processed using the MNE Python library [15]. So far, the supported raw data format is “.gdf” (GDF—general data format [37]), a standard format for EEG and biological signals. However, due to the modularity of BioPyC, Python users can easily add a new data reader as explained in Section 3.2 above.

#### 3.3.2. Preprocessing

The pre-processing is an optional step, performed using the MNE Python library as well, with multiple parameters that have to be defined through the Jupyter interface. First, users have the freedom to choose the runs and sessions they want to use for each subject. Data can be cleaned from blink artifacts using electrooculographic (EOG) channels, using MNE [15], then band-pass filtered and finally epoched based on triggers that users want to study.

#### 3.3.3. Pre-Processed Data

In this configuration, BioPyC uses data that are pre-processed and formatted, either coming from the pre-processing module presented above, or by reading a pre-processed data set using data readers. So far, our toolbox can read one type of pre-processed data format using the Python library MNE: MATLAB format, i.e., “.mat” [32]. This format was chosen since it is popular and widely used by the BCI community.

### 3.4. Applying Spatial Filters and Machine Learning Algorithms

So far, BioPyC offers algorithms that proved effective either in BCI classification competitions, notably the filter bank common spatial pattern (FBCSP) [16], standard linear discriminant analysis (LDA) and Riemannian geometry classifiers [31,38,39], or in other rapidly increasing independent fields, such of artificial intelligence, such as deep learning [40,41]. We describe them below. Finally, algorithms for physiological signals classification are presented in this section. Users can select those algorithms through the Jupyter-based GUI.

#### 3.4.1. EEG Spatial Filters

BioPyC proposes two types of spatial filters: (a) the common spatial pattern (CSP), which is widely used for binary EEG classification in BCI studies, particularly for BCIs exploiting changes in brain oscillations (also known as the frequency band power) [10]; and (b) the filter bank common spatial pattern (FBCSP) which is an improved variant of the CSP that won numerous active BCI competitions [16]. Instead of using a single frequency band, the FBCSP explores features based on spatial filters from numerous frequency bands.

#### 3.4.2. Physiological Signal Features

BioPyC offers numerous algorithms to process and extract features from each type of physiological signal, currently for heart rate (HR), breathing and electrodermal activity (EDA).

For cardiac signals, the entire electrocardiogram is first reduced to the R-R intervals (RRI) signals, where RRI corresponds to the interval between two successive heartbeats, or more precisely, the interval between two R peaks in the ECG. Note that the algorithms provided by BioPyc can be used both for ECG and for heart rate signals. Using BioPyC, one can extract the following features from cardiac signals:

- *sdRR* represents the standard deviation of the RRIs [42,43].
- *meanRR* represents the mean of the RRI [42,44].
- *RMSSD* is the root mean square of the RRIs [42,43,44].
- *CVSD* is the coefficient of variation of successive differences. This corresponds to the RMSSD divided by meanRR [42].
- *cvRR* is the RR coefficient of variation. This corresponds to the sdRR divided by the meanRR [42].
- *medianRR* is the median of the absolute values of the RRIs’ successive differences [44].
- *madRR* RRIs’ median absolute deviation (MAD) [42].
- *mcvRR* is the RRIs’ median-based coefficient of variation. This corresponds to the ratio of madRR divided by medianRR [44].
- *RR50 or RR20* is the successive RRIs’ number of interval differences greater than 50 ms or 20 ms, respectively [42].
- *pRR50 or pRR20* is the proportion derived by dividing RR50 (or RR20) by the number of RRIs [44].
- *triang* is the heart rate variability (HRV) triangular index measurement, i.e., plotting the integral of the ratio of the RRI density histogram by its height [43,45].
- *Shannon_h* is the Shannon entropy calculated on the basis of the class probabilities of the RRI density distribution [44].
- *VLF* is the HRV variance in the very low frequency (0.003 to 0.04 Hz) [42].
- *LF* is the HRV variance in the low frequency (0.04 to 0.15 Hz) [42,44].
- *HF* is the HRV variance in the high frequency (0.15 to 0.40 Hz) [42,44].
- *Total_Power* is the total power of the full density spectra [44].
- *LFHF* is the LF/HF ratio [42,44].
- *LFn* is the normalized LF power. It can be calculated using the equation “LFn = LF/(LF+HF)” [42].
- *HFn* is the normalized HF power. It can be calculated using the equation “HFn = HF/(LF+HF)” [42].
- *LFp* is the LF/Total_Power ratio [44].
- *HFp* is the HF/Total_Power ratio [44].
- *DFA* is the detrended fluctuation analysis (DFA) [46] of the heart rate raw signals.
- *Shannon* is the RRIs’ Shannon entropy [44].
- *sample_entropy* is the RRIs’ sample entropy [47].
- *correlation_Dimension* represents the RRIs’ correlation dimension [44].
- *entropy_Multiscale* is the RRIs’ entropy multiscale [47].
- *entropy_SVD* is the RRIs’ singular value decomposition (SVD) entropy [44].
- *entropy_Spectral_VLF* represents the RRIs’ spectral entropy over the VLF [44].
- *entropy_Spectral_LF* is the RRIs’ spectral entropy over the LF [44].
- *entropy_Spectral_HF* is the RRIs’ spectral entropy over the HF [44].
- *Fisher_Info* is the RRIs’ Fisher information [48].
- *Lyapunov* is the RRIs’ Lyapunov exponent [49].
- *FD_Petrosian* is the RRIs’ Petrosian’s fractal dimension [50].
- *FD_Higushi* is the Higushi’s fractal dimension of RRIs [51].

The study of the breathing signals mainly focuses on R-R intervals (RRI) for the heart rate signals and is known as the breathing rate variability (BRV). RRI corresponds to the interval between two successive breathing signals, or more precisely, the interval between two R peaks in the breathing signals. Based on these RRIs, multiple characteristics can be extracted from the signals, and are listed as follows:

- *peak_length* is the interval of successive peaks in the breathing pattern signal [52].
- *trough_length* is the interval of successive troughs in the breathing pattern signal [52].
- *peak_amplitude* is the amplitude calculated for each peak of the trial [52].
- *trough_amplitude* is the amplitude calculated for each trough of the trial [52].
- *resp_rate* corresponds to the breathing rate, obtained from the frequency domain analysis of the breathing signals [52].

Descriptive statistics can also be calculated on these characteristics, i.e., mean, standard deviation, min, max, first quartile, median and the third quartile, and be used as features to feed machine learning algorithms.

Another type of features is also recommended when manipulating breathing signals: the frequency domain-based features. To do so, BioPyC can also extract as features the power spectral density (PSD) calculated from 0.1 to 0.5 Hz, in 0.01 Hz steps.

The electrodermal activity (EDA) can be described as the superposition of two distinct skin conductance responses (SCRs): on the one hand, we have the tonic activity and on the other hand, the phasic activity [53]. In the time domain, features can be extracted from these two components through descriptive statistics, and characteristics based on deeper information can be obtained from the phasic component. These characteristics are described as follows:

- *phasic_peak_amplitude* represents the amplitude of phasic peaks [54].
- *phasic_peak_longitude* is the rise time/duration of the peaks [54].
- *phasic_peak_slope* represents the slope of the peaks [55].
- *ordinate_slope* is the ordinate of the slope of the peaks, i.e., the starting point [55].
- *peak_peak_interval* corresponds to the inter-peaks time [55].

Descriptive statistics can then be calculated on all these extracted characteristics, as well as on both tonic and phasic components, in order to define features representing the EDA signals. First, we have the basics statistics—i.e., mean, standard deviation, min, max, first quartile, median and the third quartile—that can be calculated for each of these characteristics. Second, two statistics, i.e., skewness and kurtosis, are calculated for both the tonic and the phasic components [56]. Finally, the “nb_peak_per_min” corresponds to the frequency of the phasic peaks [55] and can be defined as a feature in itself (no descriptive statistics are needed).

Concerning the frequency domain, the EDA signal spectral dynamics is largely contained in frequencies below 0.4 Hz [57]. Two types of frequency information can thus be also computed, i.e., the power spectral density (PSD) from 0.0 to 0.1 Hz, in 0.01 Hz steps [55] and the PSD from 0.045 to 0.25 Hz [58]. Altogether, BioPyC thus offers numerous features to represent, and then classify, these physiological signals.

#### 3.4.3. Machine Learning Algorithms

A user can select one or multiple classifier(s) in order to compare the classification performance on a single data set. For EEG and physiological signal classification, we chose to integrate a linear discriminant algorithm (LDA) into BioPyC, both classic and with shrinkage, since it is the most commonly used classifier in BCI studies [10,30]. The support vector machine (SVM) [59] algorithm is available as well, and can be used for EEG or physiological features classification. Then, concerning EEG signals only, we included four Riemannian classifiers [31,38]. The first ones are the minimum distance to mean with geodesic filtering classifier (FgMDM) and tangent space classifier (TSC). Such methods represent EEG signals as covariance matrices and classify them according to their (Riemannian) distances to prototypes of covariance matrices for each class (for FgMDM), or project the covariance matrices in the manifold tangent space before using Euclidean classifiers (e.g., logistic regression) in this tangent space (for TSC). Such methods recently won six international brain signals competitions [31]. In addition to those two Riemannian approaches, two new ones—filter bank FgMDM (FBFgMDM) and filter bank TSC (FBTSC)—were introduced in a recent study [39], and are also available in BioPyC. They use a bank of band-pass filters, such as the ones used for FBCSP, instead of using a unique band-pass filter, and combine Riemannian classifiers from each band. They were shown to outperform FgMDM and TSC, respectively [39]. Finally, BioPyC offers to use deep learning, which recently showed promising results for many machine learning problems, with a method from [40] called ShallowConvNet, using convolutional neural networks (CNN) dedicated to EEG classification. Moreover, due to the modularity of the toolbox, BioPyC users can easily add new classifiers (see Section 3.2), e.g., logistic regression (which is available from scikit-learn [34]) to classify data previously filtered with the CSP or FBCSP.

### 3.5. Calibration Types

**Figure 4:**
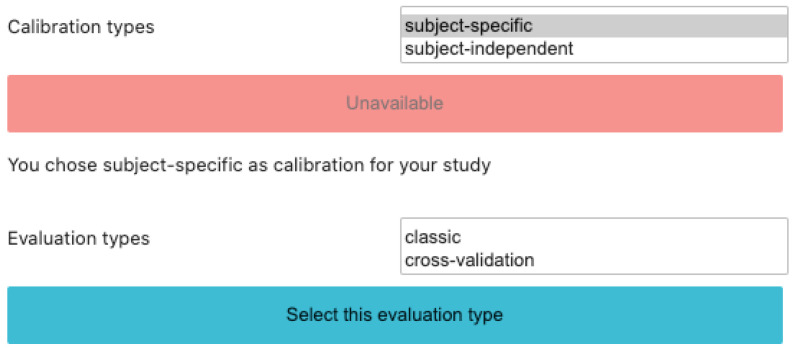
Screenshot of BioPyC’s choice of both calibration and evaluation types.

BioPyC offers users to run different types of calibration approaches for studying their data, i.e., a subject-specific calibration or a subject-independent one, as we can see on Figure 4, depending on the motivation of their experiments.

#### 3.5.1. Subject-Specific Study

So far, due to the large between-subject variability, most of the BCI studies are subject-specific, i.e., a classifier needs to be built for each individual subject [30]. First, data specific to each subject are split into two parts: the training and testing sets. Then, machine learning algorithms are trained on the first set and evaluated on the second one. To do so with BioPyC, users have to set the “split ratio” (see Section 3.6.1) through a textbox displayed on the Jupyter notebook GUI.

#### 3.5.2. Subject-Independent Study

One of the major steps for using BCIs outside the laboratories would be for BCI users to be able to instantaneously use the BCI without any calibration phase. To do so, we can evaluate machine learning algorithms through offline subject-independent studies, i.e., with a classifier built on multiple subjects and used as such on a new subject, without the need for data from this new subject. In BioPyC, the evaluation method for this type of calibration is a leave-one-subject-out cross validation, i.e., the training phase uses all subjects except the target subject data to train the classifier, and the testing phase applies this classifier on the target subject data only. This process is repeated with each subject used once as the target (test) subject.

### 3.6. Evaluation

#### 3.6.1. Split Ratio

The split ratio method for evaluation is the classic machine learning method for evaluating an algorithm. It consists in separating the data set in two parts: the first one for the training of the algorithm, and the second one for the evaluation of this algorithm. The split ratio defines the ratio of data that has to be kept for the training set. The rest of the data are used for the evaluation (test set).

#### 3.6.2. Cross-Validation

Finally, due to the usually relatively small number of trials recorded during BCIs experiments, BioPyC proposes a “leave-one-out” cross-validation method based on scikit-learn [34] for the evaluation if the number of trials is rather low. Each trial is used as a testing set where algorithms are trained on all other trials. The number of k-folds is equal to the number of trials. BioPyC proposes the “k-fold” cross-validation method as well, which allows users to choose the number of equal segments into which the data should be split. For each data segment, trials composing this segment are used as testing set when the rest of the data are used as the training set.

### 3.7. Statistics and Visualization

As explained in the introduction, BioPyC offers its users some basic statistical tests and visualizations about the classification performances obtained.

#### 3.7.1. Performances

Once algorithms are applied on the pre-processed data, classification performances scores—either the accuracy or the F1-score, depending on whether the classes are balanced or not—are automatically calculated for each subject and for each algorithm that is selected for the study. The accuracy score is calculated with scikit-learn “accuracy_score” method if the data set classes are balanced, or with scikit-learn “f1_score” method if they are unbalanced. Those classification performances are framed into a table using the Python library pandas [60], and can be directly stored into a directory that was previously indicated by the user through a Jupyter textbox, and/or used by the module “statistical analysis”, in order to make statistical testing and plotting.

#### 3.7.2. Statistics

For statistical testing, BioPyC enables users to choose to make automatic appropriate tests for comparing classification performances between machine learning algorithms with the Python libraries pandas [60] and pingouin [61]:

1. Test the data normality with the Shapiro–Wilk test from the Python library Scipy [62].
2. Test the data sphericity with Mauchly’s test from Scipy.
3. Analyze and compare means of classification performances between machine learning algorithms, using, in the case that data are normalized, the following:
  - The *t*-test: comparing the performance of two algorithms along all the subjects; comparing performances of an algorithm depending on the study type (subject-specific vs. subject-independent) using pingouin.
  - One-way ANOVA with repeated measures: comparing performances of multiple algorithms (more than two) or multiple study types using pingouin.
  - Two-way ANOVA with repeated measures: comparing performance with both factors (type of algorithms, type of study), using pingouin.

#### 3.7.3. Chance Level

When measuring classification accuracy for a BCI task, given the usually small number of samples, the actual chance level should be carefully considered [63]. For example, the chance level for a two-class paradigm will not be necessarily 50%, as it will depend on the number of testing trials and the confidence interval we want to work with. To solve this problem, BioPyC proposes an option for the calculation of the chance level based on [63]. Moreover, users can test the difference between subjects performances and the chance level with a one-sample *t*-test from [62].

#### 3.7.4. Visualization

**Figure 5:**
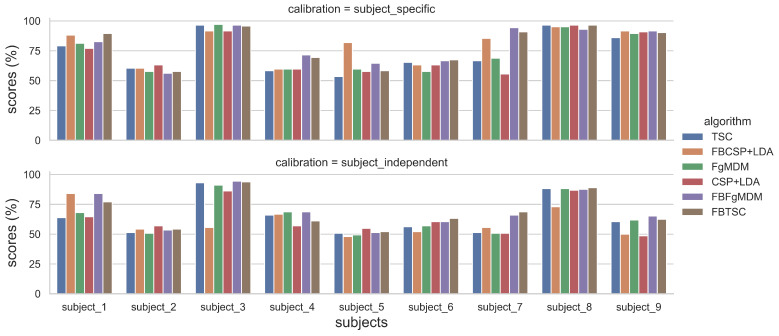
Classification accuracy of each algorithm, for each subject, on the “BCI competition IV data set 2a”, in both subject-specific and subject-independent calibrations.

**Figure 6:**
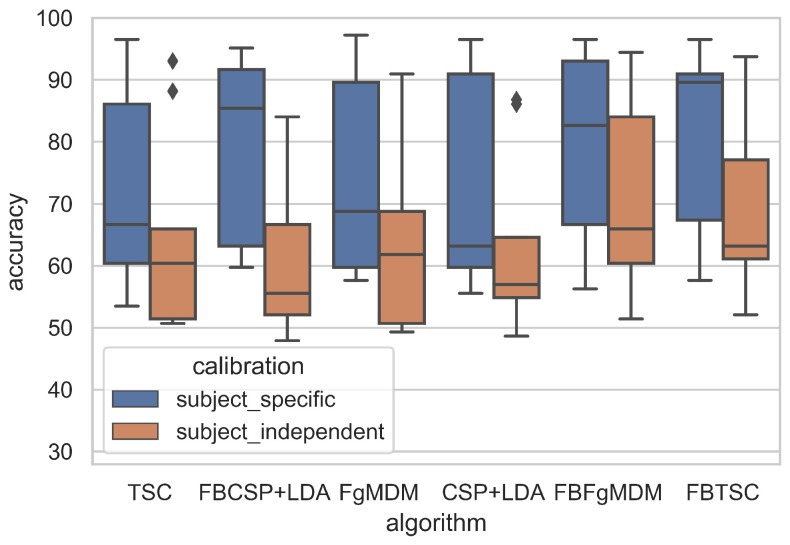
Classification accuracy of each algorithm on the “BCI competition IV data set 2a”, in both subject-specific and subject-independent calibrations.

**Figure 7:**
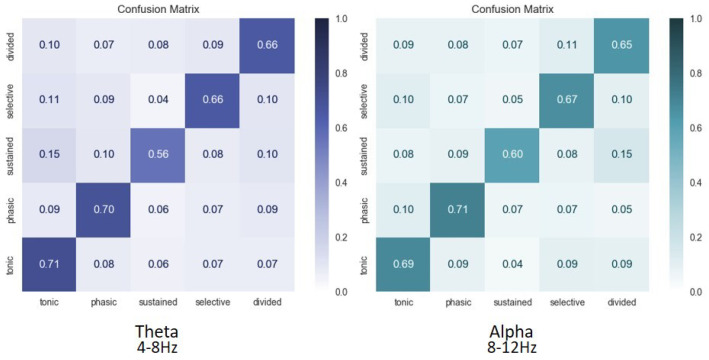
Average confusion matrices over all subjects for classification of attention in theta (4–8 Hz) and alpha (8–12 Hz) frequency bands of 5 attentional states, i.e., alertness (tonic), alertness (phasic), sustained, selective, and divided.

Data visualization can naturally be useful in BCI studies since it provides an informative and explicit feedback about the obtained classification performances. BioPyC proposes both boxplots and barplots from seaborn [64] and pairwise *t*-test visualization using scikit-learn [34]. The barplots can be used for visualizing the detailed performance results of each algorithm/calibration on each subject (see Figure 5). For the boxplots, the number of boxes on the plot will vary depending on the number of algorithms and the number of calibration types that were tested, as we can see in Figure 6. BioPyC also proposes to display confusion matrices using scikit-learn [34], as presented in Figure 7.

### 3.8. Demonstrating BioPyC Use Cases

BioPyC was already used to analyze four types of BCI data, for motor imagery BCIs and mental state decoding through passive BCIs, such as workload, emotions and attention. All data sets were of different sizes (number of subjects and trials), collected in different laboratories using different EEG devices, with data stored in different formats: this showed the versatility and robustness of BioPyC. In this section, we present the four data sets we analyzed using BioPyC.

#### 3.8.1. Motor Imagery

First, we used the modern machine learning algorithms from BioPyC to classify motor imagery EEG signals using the data set coming from [65] called “BCI competition IV data set 2a”. In this data set, EEG signals were recorded from 22 Ag/AgCl electrodes (with inter-electrode distances of 3.5 cm), from 9 subjects, when executing four different motor imagery tasks (left hand, right hand, both feet, and tongue). We chose to keep only two classes, namely the imagination of movement of the left hand (class 1) and right hand (class 2). Participants participated in two sessions of 6 runs, where a run consisted of 24 trials (12 for each class), yielding a total of 144 trials per session.

After band-pass filtering the signals in both a single band (in 8–12 Hz) single band-based algorithms, and in 94 Hz-wide bands for filter bank-based algorithms (in 4–8 Hz, 8–12 Hz, …, 36–40 Hz), we used six methods for classifying those two mental tasks, i.e., CSP coupled with a LDA [30], FBCSP coupled with a LDA [16] and 4 Riemannian approaches (FgMDM, TSC, FBFgMDM and FBTSC) [31,39] and compared them across two types of calibration, i.e., subject-specific and subject-independent. For the subject-specific calibration, classifiers were trained on the data from the first session of a subject, and tested on the data from the second session of the same subject, as done in the original study [65]. Regarding the subject-independent calibration, the training set comprised all trials of all subjects, except those of the current subject used for testing. The testing set was the second session of the current test subject.

#### 3.8.2. Workload

The second study aimed at comparing modern machine learning algorithms to classify two levels of mental workload (high versus low) based on EEG signals [39]. We used the data set coming from [66], where signals from 28 electrodes (active electrodes in a 10/20 system: F3, F7, FC3, FT7, C3, A1, CP3, TP7, P3, P7, O1, Oz, Pz, CPz, Cz, FCz, Fz, F4, F8, FC4, FT8, C4, A2, CP4, TP8, P4, P8, O2) were recorded on 22 subjects. To induce variations of the mental workload, this study used the N-back task: letters were successively displayed on the screen, and subjects had to indicate whether the current letter was the same one as the letter that displayed N letters before. The labeling was done as follows: the “low” workload corresponded to the 2 s trials from a 0-back task, while the “high” workload corresponded to the ones from a 2-back task. In total, 720 trials were recorded for each workload level and subject. The alpha rhythm (8–12 Hz) being known to vary according to workload [66], we first band-pass filtered the signals into a single band (in 8–12 Hz), before band-pass filtering them into 94 Hz-wide bands for filter bank-based algorithms (in 4–8 Hz, 8–12 Hz, …, 36–40 Hz). We then used seven methods for classifying such workload levels, i.e., the CSP coupled with a LDA [30], FBCSP coupled with a LDA [16], 4 Riemannian approaches (FgMDM, TSC, FBFgMDM and FBTSC) [31,39], as well as the ShallowConvNet from [40] and compared them across two types of calibration, i.e., subject-specific and subject independent. As described in Section 3.5, the classifiers were trained on the first half of the trials of a subject and tested on the other half for the subject-specific calibration. For the subject-independent calibration, the training set comprised all trials of all subjects, except the current subject used for testing. The testing set was the second half of the trials of the current subject.

#### 3.8.3. Affective States

The third study aimed at applying BioPyC classification algorithms to EEG signals in order to classify two types of affective states, i.e., valence (high versus low) and arousal (high versus low) [39]. Emotions are known to be difficult to detect through EEG signals in the passive BCIs field [67]. We thus challenged the recent and promising machine learning algorithms from BioPyC with a reference data set on emotions called DEAP [68]. Signals from 32 EEG electrodes (placed according to the international 10–20 system: FP1, F3, F7, FC3, FT7, C3, T7 A1, CP3, TP7, P3, P7, O1, Oz, Pz, CPz, Cz, FCz, Fz, Fp2, F4, F8, FC4, FT8, C4, T8, A2, CP4, TP8, P4, P8, O2) were recorded from 32 subjects. The data set contained 40 trials, corresponding to signals recorded when two emotion dimensions, i.e., valence and arousal, were influenced by music–video clips. Valence and arousal levels were measured using Russell’s valence–arousal scale [69] directly after each video, by clicking on a 1–9 continuous scale. This self-assessment system on a continuous scale makes the classes definition more complex: we kept the number five (corresponding to the median of the one to nine grading system) as a threshold to split trials into two unbalanced classes—low and high—for both the “emotion-arousal” and “emotion-valence” data sets.

After band-pass filtering the signals in both a single band (in 8–12 Hz, corresponding to the alpha rhythm that was proven to vary according to both valence and arousal in [67]) and in 94 Hz-wide bands for filter bank-based algorithms (in 4–8 Hz, 8–12 Hz, …, 36–40 Hz), we used seven methods for classifying both valence (high vs. low) and arousal (high vs. low) states, i.e., the CSP coupled with a LDA [30], FBCSP coupled with a LDA [16], four Riemannian approaches (FgMDM, TSC, FBFgMDM and FBTSC) [31,39], as well as the ShallowConvNet from [40] and compared them across two types of calibration, i.e., subject-specific and subject independent. For the subject-specific study, given the low number of trials, we performed a “leave-one-out” cross-validation. Thus, we used 40 models for each subject, each model being trained on 39 trials and tested on 1 trial. For the subject-independent study, we kept all trials of all subjects to compose the training set, except the current subject used for testing, as explained in Section 3.5. Note that most of the classifiers we used are able to deal with unbalanced classes, except for the CNN for which we obtained balanced classes by up-sampling the minority class by randomly duplicating trials.

#### 3.8.4. Attention

The fourth study aimed at developing a first comprehensive understanding of the different attentional states described in the model of van Zomeren and Brouwer using EEG data [70,71]. The term “Attention” encompasses several different attentional states. Given the model of van Zomeren and Brouwer it encompasses four attentional states, i.e., alertness and sustained attentions, referring to the intensity of attention (i.e., its strength), as well as selective and divided attentions, referring to its selectivity (i.e., the amount of monitored information) [72]. No study provided yet a comprehensive comparison of these different attentional states in the EEG signals.

Hence, the brain activity was recorded using BioSemi 64 active scalp electrodes (10–20 system)—AF7, AF3, F1, F3, F5, F7, FT7, FC5, FC3, FC1, C1, C3, C5, T7, TP7, CP5, CP3, CP1, P1, P3, P5, P7, P9, PO7, PO3, O1, Iz, Oz, POz, Pz, CPz, Fpz, Fp2, AF8, AF4, AFz, Fz, F2, F4, F6, F8, FT8, FC6, FC4, FC2, FCz, Cz, C2, C4, C6, T8, TP8, CP6, CP4, CP2, P2, P4, P6, P8, P10, PO8, PO4, O2—and we included 16 subjects into an experiment during which they were asked to perform different tasks. Each task assessed a type of attentional state, while we recorded the subjects’ EEG. During each task, the subjects had to react as quickly as possible to the appearance of target stimuli by pressing a keyboard space bar as quickly as possible. In accordance with the literature, the tasks and types of attention were differentiated by the type of sensory modality of the stimuli, the number of distractors, the presence of a warning tone before the stimuli and the length of the task [73,74,75,76]. For each task, 80 target stimuli were presented. We used one second prior to target presentation as the analysis window. Only data from targets that were at least one second apart from a motor response were analyzed to prevent motor-related artifacts.

First, we used BioPyC to know if we could differentiate the different attentional states from one another. We used common spatial pattern filtering in the alpha range (8–12 Hz)—-which was associated with attention in various studies [77,78]—and a linear discriminant analysis classifier, with 5-fold cross-validation.

Second, we used BioPyC to determine whether we could classify the five types of attentional states at once using only the EEG data. The subject-specific discriminability (one classifier per subject) of the EEG patterns between each of the five attention tasks was assessed, using the tangent-space classifier described in [31], with 5-fold cross-validation. We used the method from [79] to classify EEG signals into five classes: a linear discriminant analysis (LDA) was performed between each pair of class, i.e., each pair of attention tasks, and then all the resulting classifiers were combined to obtain the classification results. The 5-classes classification was performed twice with EEG data either filtered in the theta or alpha band. The confusion matrix, representing for each class the ratio of trials that were accurately or wrongfully associated with it over the total number of trials, was then computed.

## 4. Results

In this section, we present the results obtained by the different signal processing and machine learning algorithms currently offered by BioPyC.

### 4.1. Motor Imagery

The detailed results, i.e., classification accuracy scores obtained by each algorithm, for each subject, with both subject-specific and subject-independent calibrations, are represented in Figure 5. The classification accuracy distributions across subjects, for each algorithm and calibration type, are plotted on Figure 6. They revealed that FBTSC and FBFgMDM obtained the highest mean accuracy, although not significantly so, with both subject-specific (mean accuracy FBTSC = 79.6%; mean accuracy FBFgMDM = 79.7%) and subject-independent calibrations (mean accuracy FBTSC = 70.1%; mean accuracy FBFgMDM = 69.1%).

### 4.2. Workload

**Figure 8:**
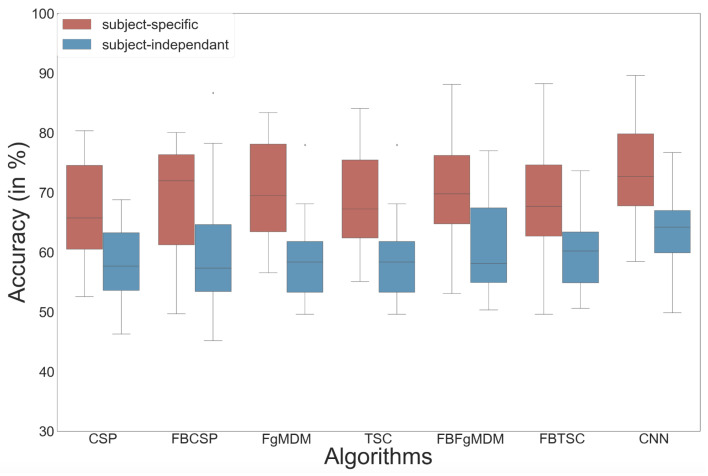
Classification accuracy of each algorithm on the workload data, in both subject-specific and subject-independent calibrations.

The results of the study are plotted in Figure 8 and reveal that the ShallowConvNet [40] obtained the highest mean accuracy, although not significantly so, with both subject-specific (mean = 72.7%) and subject-independent (mean = 63.7%) calibrations [39].

### 4.3. Affective States

**Figure 9:**
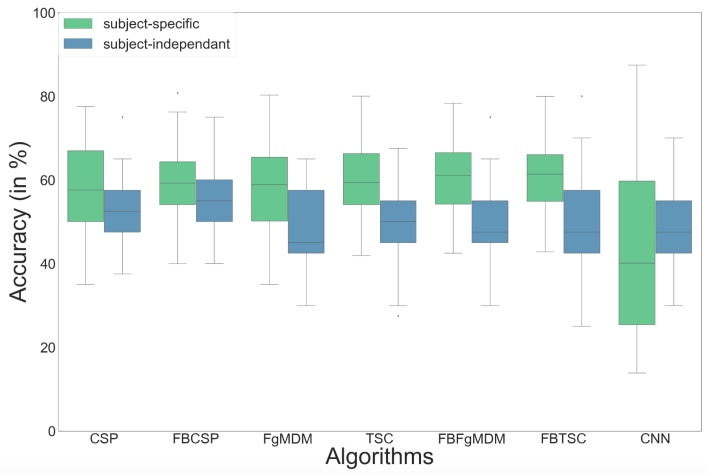
Classification accuracy of each algorithm on the valence data, in both subject-specific and subject-independent calibrations.

**Figure 10:**
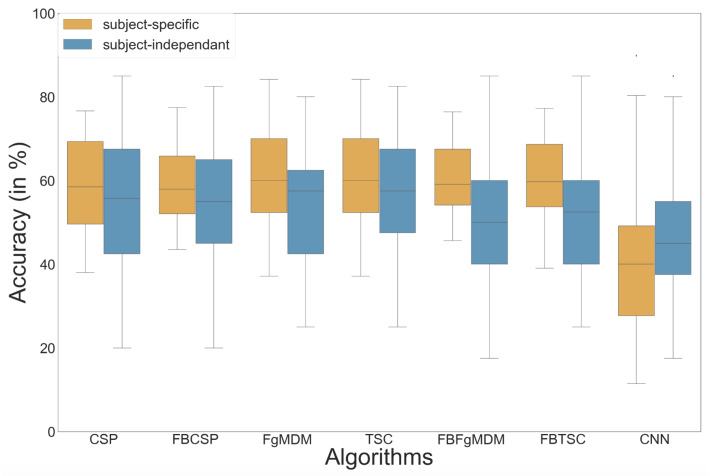
Classification accuracy of each algorithm on the arousal data, in both subject-specific and subject-independent calibrations.

The F1-score obtained for valence is reported in Figure 9, and the one for arousal in Figure 10.

Concerning the subject-specific calibration, results showed better performances for the Riemannian geometry classifiers (RGC) with the FBTSC (mean for valence = 61.0%, arousal = 60.6%) compared to state-of-the-art classification algorithms such as the CSP+LDA (valence = 57.9%, arousal = 58.1%).

In contrast, the CNN from [40] underperformed on both valence and arousal data sets (mean for valence = 46.3%, arousal = 40.1).

Regarding the subject-independent calibration, the FBCSP+LDA obtained the best performance when applied to the valence data set (mean accuracy valence = 55.23%), and the FgMDM the best one when applied to the arousal data set (mean accuracy arousal = 56.25%).

### 4.4. Attention

The results regarding the discrimination of attentional states from one another are promising and range from 83% accuracy (SD = 0.09) to discriminate alertness (tonic) from sustained attention to 74% accuracy (SD = 0.13) to discriminate selective and divided attention.

We then classified the five types of attentional states at once. The average confusion matrices over all subjects for the classification in the theta and alpha bands are displayed in Figure 7.

Overall, these promising results tend to validate the model of van Zomeren and Brouwer, as the different attentional state that they describe seem to have distinct electroencephalographic patterns of activation. We believe that future research assessing the learners’ attentional states during BCI user training might represent real opportunities to improve such training.

## 5. Discussion

In this article, we first introduced BioPyC and the different features that this software offers, before testing it through four studies, i.e., one on motor imagery, and the three others on mental states estimation. We first studied new and promising algorithms that proved efficient in recent active BCI classification competitions [16,31], such as Riemannian geometry classifiers on a motor imagery data set from [65], a cognitive workload data set [66] and an emotion data set (Valence/Arousal) [68]. Note that we also studied an algorithm that proved efficient in other fields of artificial intelligence, i.e., deep learning [40,41] on both workload and emotion data sets. We finally applied a Riemannian geometry-based method to classify five different types of attention states.

About the motor imagery data set, the filter-bank RGCs (FBFgMDM and FBTSC) obtained the best performance, although not significantly so, with both subject-specific (respectively, 79.7% and 79.6%) and subject-independent calibrations (respectively, 69.1% and 70.1%) when compared to other algorithms (FgMDMD, TSC, CSP coupled with a LDA, as well as FBCSP coupled with a LDA). Concerning the cognitive workload data set, our results suggested that a CNN obtained the highest mean accuracy, although not significantly so, in both conditions for the mental workload study—72.73% in the subject-specific study, 63.74% in the subject-independent study—outperforming state-of-the-art methods on this data set, followed by filter bank RGCs. This same CNN underperformed in both conditions for the emotion data set, a data set with small training data. On the contrary, RGCs proved to have the highest mean accuracy with the FBTSC for the subject-specific condition on the valence data-set (61.09%) and for the subject-specific condition on the arousal data set (60.60%). The classification of attentional states using single frequency bands-based RGCs showed promising results, ranging from 74% accuracy (SD = 0.13) to discriminate selective and divided attention to 83% accuracy (SD = 0.09) to discriminate alertness (Tonic) from sustained attention.

Overall, results from these four different studies highlighted the potential of RGCs methods, i.e., the filter bank RGCs—FBFgMDM and FBTSC—that proved to be efficient for motor imagery, cognitive workload and emotion classification problems, and TSC that showed interesting results when discriminating different types of attention. Indeed, FBTSC and FBFgMDM outperformed the results from other algorithms in most conditions/data sets, showing a performance gap between RGC methods using a filter bank and the ones using the single 8–12 Hz frequency band. Indeed, covering more frequency bands brings more information: it could therefore be interesting to investigate which frequency bands were chosen by the feature selection algorithms in the studies that used filter bank-based classification methods. It should be noted that a tool for analyzing and reporting which frequency bands were selected by the feature selection algorithms is already implemented in BioPyC, although it was not used for the studies presented here.

Regarding the CNN, we applied it on two data sets—workload and emotion data sets—and obtained opposite results. Indeed, the CNN obtained better performance for the workload data set, although non-significantly so, than other machine learning algorithms but underperformed on the emotion data set (for both valence and arousal). Multiple factors could explain the observed algorithm performances, such as the number of trials that are used for training models, e.g., 720 training trials for the workload data set and 39 training trials only (with cross validation calibration) for both valence and arousal data sets. This might suggest that the CNN could be useful for mental state classification, but only when large amounts of training trials are available (around 700 in our study), which is not always possible. However, other factors also differ between both data sets studied and could also explain differences in CNN performances, including the EEG epochs length (2 s epochs for workload and 60 s epochs for emotions), and the nature of the mental states studied (workload vs. emotions).

Our various experiments from the different analyses performed for BioPyC revealed several positive aspects of the software, i.e., the modularity, the comparison of classification algorithms, the statistical analyses, as well as the data visualization. First, the modularity of the software is highlighted with the different data sets formats that were used for the offline analysis presented above. Data sets for workload, emotions and attention were in a Matlab format (“.mat”) and contained pre-processed data. The data set with the motor imagery tasks was in a GDF (“.gdf”) format and required a pre-processing step before performing the signal processing and classification steps. Second, the data sets with a two-class paradigm, namely motor imagery, workload, and emotions (both valence and arousal) were used for comparing the classification algorithms, which is one of the advantages of BioPyC. Those results indicated interesting information about the compared algorithms, such as the ineffectiveness of the CNN on data sets with a small number of trials, e.g., emotions, when the same algorithms proved to be efficient on data sets with a large number of trials, e.g., workload. Moreover, BioPyC also proved to have efficient machine learning algorithms for multi-class classification. This is the case of the study on attention, where the data were divided into five classes of attention (tonic, phasic, sustained, selective and divided). Third, the automatic statistical analyses were used in two ways: (1) the two-way ANOVA with repeated measures was used for analyzing performance results of the machine learning algorithms (factor 1) with both subject-specific and subject-independent calibrations (factor 2) in the motor imagery, workload and emotions studies; and (2) post-hoc *t*-tests were performed to check significant differences in the performance results between algorithms. Fourth, concerning the BioPyC data visualization module, all plots that were shown in Section 4 were automatically generated by BioPyC: (1) boxplots represented on Figure 6, Figure 7, Figure 8, Figure 9 and Figure 10; (2) barplots represented on Figure 5; and (3) confusion matrix plots on Figure 7.

On the other hand, the studies that were run with BioPyC have pointed some limits regarding the pre-processing, e.g., EOG artifacts removal for EEG-based studies, but also regarding the processing, e.g., the lack of information about the features that were selected by the feature selection algorithms. First, concerning the pre-processing, the main missing feature is indeed an EOG artifacts removal system: EEG signals are known to be sensitive to noise, and multiple methods are available to reduce this noise. For example, tools such as artifact rejection based on ICA are available in Python libraries, such as MNE [15]. About the processing, machine learning algorithms obtained interesting classification performances among these four studies. However, filter-bank RGCs, i.e., FBTSC and FBFgMDM, proved to be robust by obtaining the best classification performance on the motor imagery and the emotion (valence/arousal) data sets, as well as the second best classification performances on the workload data set, but we did not check which frequency bands were used for each data set to obtain such results. We indeed chose to apply single band-based algorithms such as the CSP coupled with a LDA on the alpha band (8–12Hz) for most of the studies, but more information has probably been given by other frequency bands (theta or beta) when using Filter bank RGCs. Thus, we implemented a first module to report the frequency bands selected by the feature selection algorithm (not shown in this paper). However, in general, it would be interesting to implement, in the future, machine learning introspection algorithms to understand and interpret what the machine learning algorithms have learned from the data [80]. Still regarding the processing step of BioPyC, a module for physiological signals classification, e.g., EDA, respiration and ECG, was implemented in BioPyC. While we did not report on their use in this paper, they were tested in a recent study for estimating the curiosity levels in brain and physiological signals [81]. It should therefore be relevant to run several other studies in order to further validate the methods that are proposed for each of these physiological signals. The classification of such physiological signals usually requires extracting many features from them, and a feature selection algorithm is generally applied in order to keep the features that bring the most interesting information to the machine learning algorithms. As for the filter bank methods that were used for classifying EEG signals in our studies, it would therefore be useful to study the features that are extracted and selected from the physiological signals. Finally, another limit of BioPyC is the absence of modules, which would study the electrodes that bring the most information to the machine learning algorithms and, therefore, the brain areas that are mostly involved in the various studies conducted.

## 6. Current Status and Future Work

BioPyC is currently publicly available on GitHub at https://gitlab.inria.fr/biopyc/BioPyC/; accessed on 25 August 2021. Users have to clone the repository and install all dependencies (Jupyter, voilà, numpy, pandas, pingouin, pyRiemann, scikit learn, scikit_posthocs, MNE 0.17 and braindecode) using pip. Then, users have to find the file BioPyC.ipynb and run “voila BioPyC.ipynb” in order to display the interface in a web browser and initialize the application. All instructions will then be given by the application that is made as an intuitive tutorial.

In its current stage, BioPyC offers the different modules that allow users to follow the standard steps of the BCI process, i.e., reading different EEG data format, filtering and cleaning EEG signals, classifying EEG signals and finally visualizing and performing statistical tests on the classification performance results.

Regarding the reading of different EEG formats, two modules are available in the current stage of the platform, namely GDF (“.gdf”) and Matlab (“.mat”), and one is still in progress, i.e., MNE (“.fiff”). Future versions on BioPyC will offer more modules for reading data, starting with those for Python, i.e., “.pkl” and “.dat”.

BioPyC currently offers tools for pre-processing the signals as well, all based on MNE [15]: band-pass filtering and epoching. More pre-processing features are available in MNE, such as EOG-based artifacts removal. It would, therefore, be easy to add new modules for pre-processing data in the future versions of BioPyC. Indeed, EEG signals being noisy, artifact correction is an important part of the pre-processing step of the BCI process. This feature will, therefore, be the next future addition to BioPyC.

Regarding the third step of the BCI process—i.e., signal processing and machine learning for EEG signals classification—so far, BioPyc proposes several efficient algorithms for decoding oscillatory activity: the CSP [30] and FBCSP [16] for spatial filtering, and the LDA, Riemannian geometry methods [31,39], as well as the CNN [40] as machine learning algorithms implemented in BioPyC. However, the ongoing works aim to integrate new signal processing and machine learning algorithms for the classification of event related potentials (ERPs) into BioPyC. Among them, xDAWN [82], which is widely used for spatial filtering and has proved to be efficient for EEG-based classification of workload levels [83]. It would also be interesting to integrate other ERP spatial filtering methods into the future versions of BioPyC, e.g., principal component analysis (PCA) or canonical correlation approaches (CCA) [84] that proved efficient for EEG classification of mental workload levels as well [85]. Moreover, current works also aim at integrating machine learning methods for the classification of both ERPs and oscillatory activity with BioPyC, e.g., EEGNet [86]. Finally, the fourth and last step of the BCI process, i.e., performing visualization and statistical tests on classification performance results, also benefits from current improvements: a new module for visualizing the percentage of use of the different frequency bands selected by the filter bank-based algorithms, i.e., FBCSP, FBFgMDM and FBTSC, is now integrated but has not been used so far. Moreover, a module for analyzing which brain areas are used by the classifiers (i.e., which electrodes brought the most information) will be soon integrated into BioPyC.

## 7. Conclusions

We presented BioPyC, an open-source and easy-to-use BCI Python software for offline EEG and biosignal analysis. This platform allows BCI and physiological computing researchers to quickly analyze offline their data by following the classical steps of pre-processing (optional), signal processing and classification, statistical analysis and data visualization. It is important to note that users do not need any programming skills to be able to process their data since BioPyC is built in the form of a Jupyter notebook with a voilà GUI that acts as a tutorial: each step is described with instructions that guide users in their analyses and choices for parameters and algorithms to use. BioPyC is already proved to be a comprehensive tool since it was used for four extensive studies so far, with quite different aims and requirements. Moreover, since Python is free of charge, any researcher can use it for his/her experiments. Moreover, BioPyC is open source and allows users to build new modules. For example, new signal processing or classification algorithms can be easily added to the platform, as well as data readers for new data sets formats. So far, BioPyC has still a modest number of tools but can easily be extended in the future and is still growing. For example, currently, the toolbox can only support one main BCI paradigm, i.e., oscillatory-based BCI, but will soon be extended to support the evoked potentials-based BCIs paradigm.
